# Lysophosphatidic Acid Upregulates Laminin-332 Expression during A431 Cell Colony Dispersal

**DOI:** 10.1155/2010/107075

**Published:** 2010-08-25

**Authors:** Hironobu Yamashita, Manisha Tripathi, Jerome Jourquin, Yoonseok Kam, Shanshan Liu, Brandy Weidow, Vito Quaranta

**Affiliations:** ^1^Department of Cancer Biology, Vanderbilt University Medical Center, Nashville, TN 37232, USA; ^2^Integrated Mathematical Oncology Department, H. Lee Moffitt Cancer Center, Tampa, FL 33612, USA

## Abstract

Lysophosphatidic acid (LPA) is a bioactive phospholipid that affects various biological functions, such as cell proliferation, migration, survival, wound healing, and tumor invasion through LPA receptors. Previously, we reported that LPA induces A431 colony dispersal, accompanied by disruption of cell-cell contacts and cell migration. However, it remains unclear *how* LPA affects cell migration and gene expression during A431 colony dispersal. In this paper, we performed cDNA microarray analysis to investigate this question by comparing gene expression between untreated and LPA-treated A431 cells. Interestingly, these results revealed that LPA treatment upregulates several TGF-*β*1 target genes, including laminin-332 (Ln-332) components (*α*3, *β*3, and *γ*2 chains). Western blot analysis also showed that LPA increased phosphorylation of Smad2, an event that is carried out by TGF-*β*1 interactions. Among the genes upregulated, we further addressed the role of Ln-332. Real-time PCR analysis confirmed the transcriptional upregulation of all *α*3, *β*3, and *γ*2 chains of Ln-332 by LPA, corresponding to the protein level increases revealed by western blot. Further, the addition of anti-Ln-332 antibody prevented LPA-treated A431 colonies from dispersing. Taken together, our results suggest that LPA-induced Ln-332 plays a significant role in migration of individual cells from A431 colonies.

## 1. Introduction

Lysophosphatidic acid (LPA) is a phospholipid growth factor involved in a variety of physiological functions such as cellular proliferation, differentiation, migration, and survival [1, 2]. In addition, it has also been shown to have a role in pathological conditions like wound healing and tumorigenesis [3]. LPA is naturally produced by many cells, including platelets, fibroblasts, and adipocytes; therefore, body fluids like serum, saliva, and other follicular fluids are a rich source of LPA [4–7]. It is well established that LPA signals various events through its G protein-coupled receptors (GPCR), namely, LPA-1-4 [8]. 

During tumorigenesis, LPA is reportedly involved in both cell migration and invasion [9–12]. Many reports have shown that LPA plays an important role particularly in ovarian cancer, affecting various aspects of cancer development, including cellular proliferation, angiogenesis, migration, and survival [13, 14]. It has also been shown that increased levels of LPA are found in the plasma of ovarian cancer patients, compared to disease-free subjects [15]. Normal ovarian epithelial cells produce trace amounts of LPA, whereas ovarian cancer cells produce LPA in large amounts [16]. Similarly, in prostate cancer, LPA is reported to induce proliferation and survival of androgen-independent prostate cancer cells [17, 18]. In lung cancer, LPA has been shown to induce cellular migration [19]. LPA levels are also significantly increased in patients with myeloma, endometrial cancer, and cervical cancer [20, 21]. Autotaxin, a soluble exoenzyme upregulated in cancer cells that increases cell invasion, has been found to stimulate proliferation and motility of cancer cells through LPA production [22], whereas autotaxin-dependent motility is blocked in LPA-1 deficient fibroblasts [23].

Previously, we have shown that LPA causes dispersal of epithelial cell colonies [24]. Our report demonstrated stepwise upregulation of cell motility features after treatment of A431 cells with LPA. Specifically, we noted the following changes: stimulation of lamellipodia formation, downregulation of cell-cell adhesion, and enhanced migration of the cells. In continuing this line of research, we have thus hypothesized that LPA changes cell motility by somehow affecting the cell microenvironment. To begin to test this hypothesis, in this study we performed microarray analysis of A431 cancer cells treated with LPA or PBS (for control) to examine the changes in microenvironment-related genes. Interestingly, microarray results revealed that LPA upregulates several TGF-*β*1 target genes along with Ln-322, a major component of basement membrane. Several independent approaches also supported this finding.

## 2. Materials and Methods

### 2.1. Reagents

L-*α*-lysophosphatidic acid (oleoyl, LPA 18 : 1) was purchased from Avanti Polar Lipids (Alabaster, AL) and stored at −80°C. Polyclonal antibody (pAb) against phosphorylated Smad2 (Ser465/467), GAPDH, and monoclonal antibody (mAb) against Smad2/3 were purchased from Cell Signaling Technology (Danvers, MA), BD Biosciences (San Jose, CA), and Invitrogen (Carlsbad, CA), respectively. BM165 (mAb against human laminin *α*3 chain) was purchased from Life Technology Invitrogen (Carlsbad, CA). H-300 (pAb against human laminin *β*3 chain) was purchased from Santa Cruz Biotechnology (Santa Cruz, CA). 2778, pAb against laminin *γ*2 chain was prepared in-house, as previously described in [25, 26]. MAb against *β*-actin was purchased from Sigma (St. Louis, MO). Anti-rabbit IgG or mouse IgG HRP-conjugated antibody was purchased from GE health care (Pittsburgh, PA).

### 2.2. Cell Culture

A431, a human epidermal squamous carcinoma cell line, was obtained from American Type Culture Collection (Manassas, VA), maintained in DMEM (Invitrogen), supplemented with 10% fetal bovine serum (FBS; Invitrogen) and 2 mM L-glutamine, and kept in constant culture in a humidified incubator with 5% CO_2_ at 37°C.

### 2.3. Immunoblot Analysis

To examine phosphorylation of Smad2 and expression of Ln-332 chains by LPA, A431 cells were serum starved for 24 h and incubated with PBS or LPA (1–4 *μ*M) for indicated periods of time. Cells were lysed and 100 *μ*g of proteins were separated on 4–12% NuPAGE (Bis-Tris) gels under reducing conditions. 3T3NIH cell lysates were purchased from Cell Signaling and used as a positive control (for Smad2 phosphorylation). After separation, the same gel was transferred to a PVDF membrane and blocked with 5% skim milk in 1X TBS and 0.1%Tween 20. After the blocking step, pAb against phosphorylated Smad2 (pSmad2; Ser465/467), GAPDH, or mAb against Smad2/3 was added at the diluted ratio of 1 : 1000 and incubated at 4°C overnight. To check the expression of laminin *α*3, *β*3, and *γ*2 chains, BM165 mAb, H-300 pAb, or 2778 pAb was added at the diluted ratio of 1 : 1000, 1 : 200, 1 : 2000, respectively. MAb against *β*-actin was used as an internal control at the diluted ratio of 1 : 10000. Anti-rabbit IgG or mouse IgG HRP-conjugated antibody was used as a secondary antibody at the diluted ratio of 1 : 1000 for 1 hr at room temperature. The bands in all blots were visualized and imaged with an ECL plus system (Perkin Elmer, Waltham, MA). Band densities were quantified using *ImageJ*. Differences were examined with Student's *t*-test statistics using SPSS, version 17 (Chicago, IL), with *P* values less than  .05 accepted as significant.

### 2.4. RNA Extraction and cDNA Synthesis

To detect the expression level of laminin *α*3, *β*3, and *γ*2 chains in A431 cells, total RNA was isolated from cells treated with PBS or 1-2 *μ*M (1 *μ*M for microarray analysis and 2 *μ*M for real-time PCR) LPA using an RNAeasy kit (Qiagen, Germantown, MD). Total RNA digested with RQ RNase-free DNase I (Promega, Madison, WI) and OligoT primer (Roche, Madison, WI) was incubated for 10 min at 70°C, chilled on ice, and added to a 20 ml aliquot of a reaction mixture containing 4 *μ*l of Transcriptor RT reaction buffer (Roche), 1 *μ*l of 10 mM mixture of four dNTPs, and 1 *μ*l of RNasin (Promega). In addition, 0.5 *μ*l of Transcriptor Reverse Transcriptase (Roche, Madison, WI) was added and incubation continued for 1 h at 55°C. To stop the reaction, the reaction mixture was heated for 5 min at 85°C and then chilled on ice. To remove RNA contamination in the reaction mixture, RNase H (Promega) was added and incubated for 30 min at 37°C. RNA integrity was determined by electrophoresis on a 1% agarose gel followed by visualization of intact 18S and 28S ribosomal RNA bands. RNA purity was measured using the *A*_260_/*A*_280_ ratio. To ensure that the optical density at *A*_260_ was in the linear range, various concentrations of RNA were plotted against absorbance.

### 2.5. cDNA Microarray Analysis

Microarray experiments were performed by the Vanderbilt University Medical Center Microarray Core using Affymetrix Human U133 plus 2.0 chips according to manufacturer's instructions. Data were normalized using the Microarray Suite 5.0 algorithm. Due to our interest in particular genes, only some probe sets were analyzed, as shown in Table 1.

Comparisons were made between RNA extracted from PBS-treated A431 cells and LPA-treated A431 cells (*N* = 2, in triplicate). Differences were examined with Student's *t*-test statistics using SPSS, version 17 (Chicago, IL), with *P* values less than  .05 accepted as significant.

### 2.6. Quantitative Real-Time PCR (RT-PCR)

For RT-PCR, we analyzed samples on a MiQ machine (Bio-Rad Laboratories, Hercules, CA) using a FastStart SYBR Green Master Mix (Roche, Madison, WI). The following primer sets were used, as shown in Table 1: LAMA3 (forward): 5′-GGCTCACTCTGTATTGTTGG, LAMA3 (reverse): 5′-ACAGAGACTGCTTTGGTGTG, LAMB3 (forward): 5′-TGATGGACAGGATGAAAGAC, LAMB3 (reverse): 5′-GGAAGCTGTAGCATCACTTG, LAMC2 (forward): 5′-GAAGCCCAGAAGGTTGATAC, LAMC2 (reverse): 5′-GTGAGTGTTCTGGAGCAAAG, GAPDH (forward): 5′-ATGACATCAAGAAGGTGGTG, GAPDH (reverse): 5′-CTGTAGCCAAATTCGTTGTC. PPIA (forward): 5′-CAAATGCTGGACCCAACACA, PPIA (reverse): 5′-TGCCATCCAACCACTCAGTC. These primers were designed by open-source Primer3 software (http://primer3.sourceforge.net).

Comparisons were made between RNA extracted from PBS-treated A431 cells and LPA-treated A431 cells (*N* = 2, in duplicate or triplicate). Differences were examined with Student's *t*-test statistics using SPSS, version 17 (Chicago, IL), with *P* values less than  .05 accepted as significant.

### 2.7. Cell Dispersal Assays

For dispersal assays, A431 cells were seeded at a density of 10^4^ cells/ml in 6-well plates and allowed to grow for 24 h. After formation of colonies, cells were cultured in serum-free DMEM for 24 h and then treated with PBS or LPA for 4 h. Microscopy was conducted using a Zeiss Axiovert 200 M microscope (Zeiss, Thornwood, NY) equipped with a temperature- and CO_2_-controlled chamber (5 images per well, triplicate wells per treatment). Microscopy was under the control of OpenLab software (Improvision, Lexington, MA). To quantify cell behavior, we manually counted both the number of dispersed colonies and total colonies in each well of microplates. Differences were examined using two-way ANOVA with Bonferroni posttest using GraphPad Prism5 (La Jolla, CA), with values less than  .05 accepted as significant. Data are presented as the mean ± standard deviation ratio (percentage) of dispersed colonies to total number of colonies imaged. 

## 3. Results and Discussion

**Figure 1:**
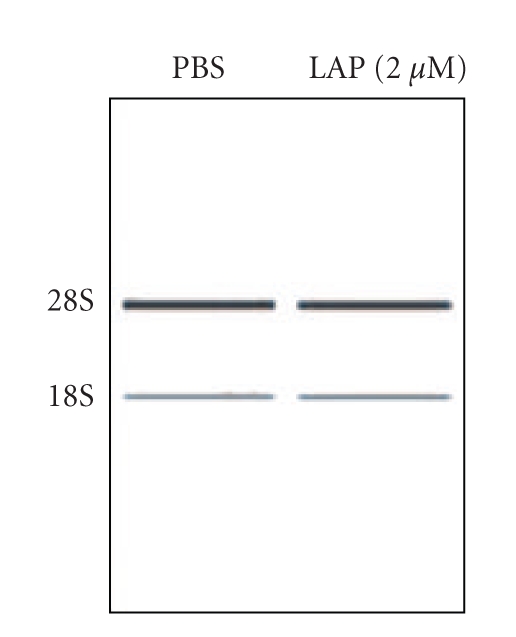
*RNA preparation.* A431 cells were incubated for 24 h in serum-deprived culture medium. LPA (2 *μ*M) or PBS (control) was added to medium and incubated for 12 h at 37°C in a humidified, 5% CO_2_, 95% air atmosphere. Total RNA was isolated from A431 cells treated with PBS or LPA (2 *μ*M) for 12 hrs, using a Qiagen RNeasy kit. The RNA integrity was measured by the RNA integrity number (RIN) software algorithm designed to classify the eukaryotic total RNA, based on numbering from 1 to 10 (1 indicates the most degraded RNA profile, and 10 indicates the most intact RNA). Electrophoresis of RNA in eukaryote total RNA Nano_DE114000902 resulted in an RNA integrity number (RIN) of 10.

We previously reported that LPA dramatically induces A431 squamous carcinoma cell colony dispersal, accompanied by disruption of cell-cell contacts and individual cell migration [24]. Herein, we analyzed gene expression of A431 cancer cells treated with LPA in order to better understand how LPA functions during cell colony dispersal. To this end, we first recapitulated experiments from our previous studies, whereas A431 cells were serum starved for 24 h and then treated with LPA (1 *μ*M) or PBS (for control) for 12 h. After incubation, A431 cells were collected, and total RNA was extracted for cDNA microarray analysis. The RNA integrity was measured by the RNA integrity number (RIN) software algorithm designed to classify the eukaryotic total RNA, based on numbering from 1 to 10 (1 indicates the most degraded RNA profile, 10 indicates the most intact RNA). The RNA integrity number was checked by electrophoresis of RNA in Eukaryote Total RNA Nano_DE114000902. The RIN results of total RNA treated with PBS or LPA showed the number of 10, showing that both RNA samples are intact RNA (Figure 1). 

**Figure 2:**
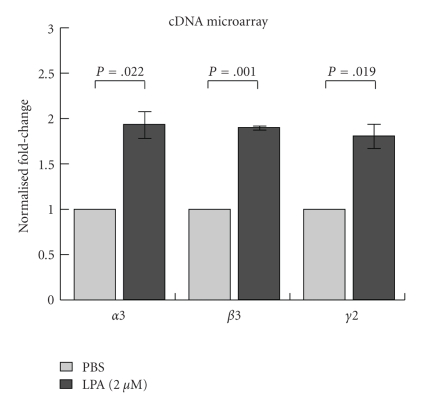
*cDNA microarray analysis. *Microarray analysis was performed on PBS and LPA-treated samples using Affymetrix Human U133 plus 2.0 chips according to manufacturer's instructions, and data were normalized using the Microarray Suite 5.0 algorithm. Among the upregulated genes induced by LPA, all three subchains of the intact Ln-332 molecule: *α*3 chain (LAMA3), laminin *β*3 (LAMB3), and laminin *γ*2 chain (LAMC2) were significantly increased.

Affymetrix cDNA microarray analysis was then performed to compare gene expression of A431cells treated with either LPA or PBS. Due to our interest in particular genes, only some probe sets were processed and analyzed for this study. Of note, laminin *α*3 (LAMA3), *β*3 (LAMB3), and *γ*2 chains (LAMC2), all components of the Ln-332 heterotrimer, were significantly upregulated in LPA-treated samples (Figure 2; *N* = 2, in duplicate or triplicate; *P* =  .022, .001, and  .019, resp.). These results also revealed that several other genes involved in extracellular matrix were overexpressed in LPA-treated samples, including tenascin C, cysteine rich protein 61, thrombospondin-1, and serine peptidase inhibitor or plasminogen activator inhibitor-1 (data not shown). Interestingly, all of these genes have been shown to be TGF-*β*1 target genes [27–31]. 

**Figure 3:**
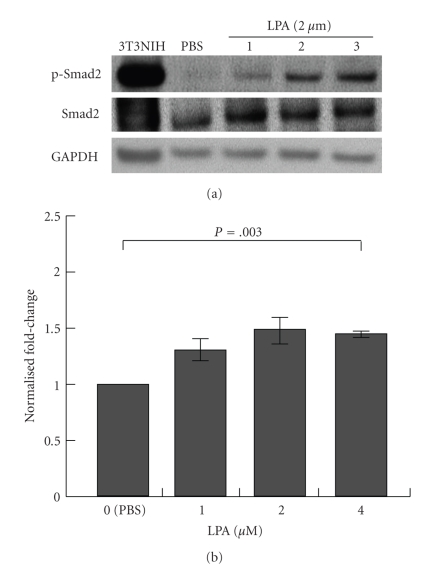
*LPA induces phosphorylation of Smad-2 during A431 colony dispersal.* A431 cells were serum starved for 24 h and incubated with PBS or LPA (1, 2, or 4 *μ*M) for 1 h. (a, b) Cells were lysed and 100 *μ*g of total proteins were separated on 4–12% NuPAGE gels under reducing conditions. 3T3NIH cell lysate after TGF-*β*1 treatment was used as a positive control for phosphorylation of Smad2. After separation, the same gel was transferred to a PVDF membrane and blocked with 5% skim milk in 1X TBS and 0.1% Tween 20. After blocking, a pAb against phosphorylated Smad2 (p-Smad2; Ser465/467), a mAb against Smad2/3, or GADPH was added at the diluted ratio of 1 : 1000 and incubated at 4°C overnight. Anti-rabbit IgG or mouse IgG HRP-conjugated antibody was used as a secondary antibody at a diluted ratio of 1 : 1000. The bands were visualized with an ECL plus system. Protein expression levels were quantified from the western blots using *ImageJ*. Results showed that 4 *μ*M LPA treatment for 12 h significantly enhanced pSmad2 protein expression compared to PBS treatment (*N* = 3; *P* =  .003).

The original observation on A431 colony dispersal has led us to further investigate cell phenotype epithelial-to-mesenchymal transition (EMT), which is reportedly induced by TGF-*β*1 in some cell systems and shows a disconnect between cells and dramatic changes in gene expression [32, 33]. TGF-*β*1 is a protein that controls cell proliferation and growth in many cells, stimulates production of extracellular matrix proteins, and induces EMT, which is accompanied by the decrease of cell-cell contacts and the increase of cell motility [24]. TGF-*β*1 is known to bind and activate TGF-*β*1 type I receptor (TGF*β*R1), which phosphorylates receptor-regulated Smad (R-Smad), Smad2, and Smad3 [34]. Phosphorylated Smad2/3 interacts with Smad4 to form complexes that translocate into cell nuclei, which leads to regulation of gene expression, either negatively or positively [34]. Recently, there were several reports suggesting the crosstalk between GPCR activated by LPA or sphingosine 1-phosphate and TGF*β* signaling. For example, in keratinocytes, it has been reported that LPA induces TGF-*β*1 signaling to mediate growth arrest and chemotaxis [35]. Therefore, we performed western blot analysis to determine whether LPA induces the phosphorylation of Smad2 in A431 cells, using an anti-phospho-Smad2 antibody (3A). These results showed that, in contrast to the PBS control, LPA induced phosphorylation of Smad2 in A431 cells, although, of the concentrations we tested, only the highest dose, 4 *μ*M, significantly enhanced phosphorylation (Figure 3(b)). Our results are in line with previous studies by Jeon et al. [36], which demonstrated that Smad2/3 is phosphorylated upon LPA stimulation and that it can be blocked using an LPA antagonist Ki16425. These findings suggest that LPA may also induce TGF-*β*1 signaling in A431 cells. 

**Figure 4:**
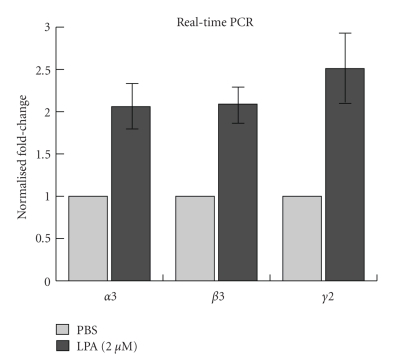
*RT-PCR analysis of mRNA expression of laminin* *α* *3,* *β* *3, and* *γ* *2 genes in LPA-treated A431 cells. * To confirm the results of the previously performed microarrays, RT-PCR analysis was performed as described in Section 2. Briefly, A431 cells were treated with PBS or 2 *μ*M LPA for 12 h, total RNA was extracted for each sample, and cDNA was synthesized using reverse transcriptase. Samples were analyzed on a MiQ machine using FastStart SYBR Green Master Mix. The primer sets used are shown in Table 1. Results showed that LPA treatment increased Ln-332 component, *α*3, *β*3, and *γ*2, expression (by ~2–2.5-fold), although results were not determined to be significantly different than PBS control treatments (*N* = 3; *P* =  .11,  .08, and .13, resp.).

Among the overexpressed genes induced by LPA, we further investigated the three subcomponents of the intact Ln-332 molecule: *α*3 chain, *β*3, and *γ*2 chains. Ln-332 is major basement membrane glycoprotein expressed mainly in epithelial cells that consists of these three chains in the form of heterotrimer *α*3*β*3*γ*2 [36, 37]. Ln-332 plays a significant role in controlling cell behavior, such as cell adhesion, migration, and spreading via cell surface receptors, including integrin *α*3*β*1 and integrin *α*6*β*4 [37, 38]. We performed quantitative RT-PCR analysis using laminin primer sets shown in Table 1 to confirm the upregulation of the three laminin genes upon LPA treatment of A431 cells,. Real-time PCR analysis showed that LPA somewhat increased the expression of laminin *α*3, *β*3, and *γ*2 chains in A431 cells (by ~2–2.5-fold compared to PBS controls), although not significantly (*N* = 3; *P* =  .11,  .08, and  .13, resp.), suggesting that LPA may also induce laminin *α*3, *β*3, and *γ*2 gene expression at the mRNA level (Figure 4). However, additional studies are needed to confirm this finding.

**Figure 5:**
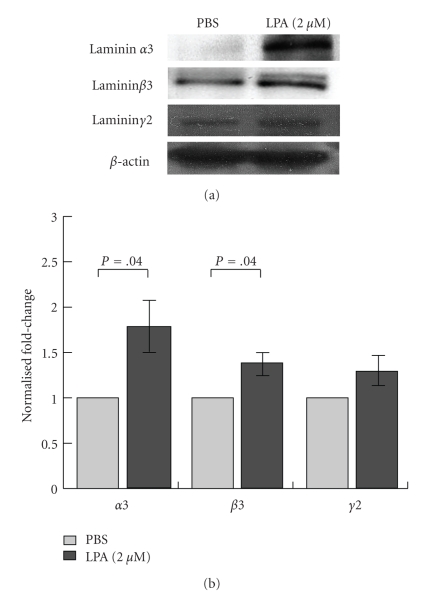
*LPA enhances laminin-332 protein expression in A431 cells.* To examine the expression level of Ln-332 chains in LPA-treated A431 cells, cells were serum starved for 24 h and incubated with PBS or 2 *μ*M LPA for 12 h. (a, b) Cells were then lysed and 100 *μ*g of proteins were separated on an 8% gel under reducing conditions. After separation, the same gel was transferred to a PVDF membrane and blocked with 5% skim milk in 1X TBS and 0.1% Tween 20. After blocking, BM165 (mAb against human laminin *α*3 chain) H-300 (pAb against human laminin *β*3 chain), a pAb against rat laminin *γ*2 chain, or a mAb against actin was added at the diluted ratios of 1 : 1000, 1 : 200, 1 : 2000, and 1 : 10000, respectively, and incubated at 4°C overnight. Anti-rabbit IgG or mouse IgG HRP-conjugated antibody was used as a secondary antibody at the diluted ratio of 1 : 1000. The bands were visualized with an ECL plus system (Perkin Elmer, Waltham, MA). Protein expression levels were quantified using *ImageJ*. Results showed that LPA treatment consistently increased Ln-332 component expression. Both the Ln-332 *α*3 and *β*3 chains were determined to be significantly increased (*N* = 3, *P* =  .004), whereas the *γ*2 chain was only somewhat elevated compared to the PBS control treatment.

Furthermore, western blot analysis using antibodies against individual laminin *α*3, *β*3, and *γ*2 chains also showed that protein expression increased after LPA treating of A431 cells. Ln-332 *α*3 and *β*3 chains were significantly enhanced in LPA-treated cells, whereas *γ*2 chain was only somewhat increased using this method (Figures 5(a) and 5(b)). Collectively, these results suggest that Ln-332 heterotrimer expression is also elevated after LPA treatment. Interestingly, various cytokines, growth factors, and LPA have previously been shown to promote the synthesis of Ln-332 in human keratinocytes [39]. Taken together, three independent approaches show that Ln-332 is enhanced at the cDNA, mRNA, and protein levels, respectively. 

**Figure 6:**
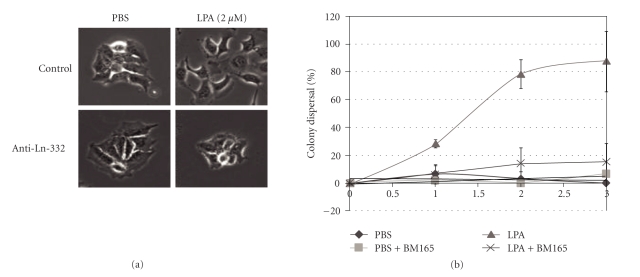
*Anti-Ln-332 antibody prevents A431 colony from dispersing by LPA treatment.* Cell colony dispersal assays were performed similarly to our previous studies (serum-starved A431 cells for 24 h, treated with 2 *μ*M LPA), with the addition of an anti-Ln-332 antibody (BM165). (a, b) We found that anti-Ln-332 antibody significantly blocked dispersal of LPA-treated colonies, compared to those colonies treated with LPA alone (*N* = 3; *P* <  .05). LPA-treated A431 colony dispersal percentages in the absence (LPA) or presence of BM165 antibody (LPA + anti-Ln-332) are indicated as light-grey triangles or cross, respectively. These results strongly support a general role for Ln-332 in regulating the “LPA effect”. *P <  .05, Two-way ANOVA (Bonferroni post test).

The binding and their role of Ln-332 and integrin receptors have been intensely studied in various physiological events [43]. Ln-332 can promote strong cell adhesion by interacting with *α*6*β*4 and intermediate filaments to form hemidesmosomes [37, 44–46] or can behave as a promigratory agent by binding to *α*3*β*1 [47, 48]. These functions are observed in normal skin and wound healing, respectively [37, 44, 45, 48]. Collectively, these findings lead us to believe that LPA-inducible Ln-332 promotes A431 cell colony dispersal, presumably by interaction with cell surface receptor integrins *α*3*β*1 or *α*6*β*4. While the experimentation necessary to unravel this hypothesis is still in early stages, we have obtained some data that supports this idea. Using cell colony dispersal assays similar to our previous studies [24], we have found that adding anti-Ln-332 antibody to A431 cells almost completely blocked the dispersal of LPA-treated colonies (Figures 6(a) and 6(b)). These results strongly support a general role for Ln-332 in regulating the “LPA effect”. In addition, preliminary studies have also shown that blocking antibodies against integrins *α*3 or *β*1, but not *α*6, remarkably reduced LPA-treated A431 colony dispersal (data not shown). Our findings are in line with previous studies by Salo et al. [49], which showed that antibodies blocking Ln-332 inhibit cell adhesion and migration, subsequently leading to reduced tumor growth and invasion *in vivo*. However, we plan to tackle additional studies in the future to further investigate the specific role of LPA in these data. Of note, Giannelli et al. previously demonstrated that Ln-332 and TGF-*β*1 cooperatively induce EMT in hepatocellular carcinoma [50]. 

**Figure 7:**
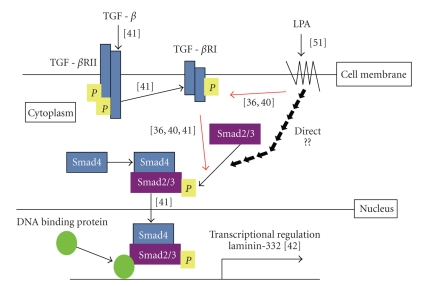
*Hypothetical working model of LPA-induced A431 colony dispersal. * We hypothesize that LPA induces Ln-332 expression via TGF-*β*1 pathway. In our working model, LPA binds to LPA receptors on the A431 cell surface and transactivates TGF-*β*1 type I receptor (TGF*β*R1), which phosphorylates receptor-regulated Smad (R-Smad), Smad2, and Smad3. These results are supported by other previous findings [36, 40, 41]. Another potential pathway may lead to the direct induction of phosphorylation of Smad2/3.Phosphorylated Smad2/3 forms complex with Smad4 and translocates into the nucleus [41]. Smad4 functions as a positive transcriptional regulator of Ln-332 [42]. Finally, LPA enhances Ln-332 expression to promote A431 colony dispersal.

In summary, we propose a working model of LPA-induced A431 colony dispersal (Figure 7). We hypothesize that LPA induces Ln-332 expression via the TGF-*β*1 pathway. In our working model, LPA binds to LPA receptors on the A431 cell surface and transactivates TGF-*β*1 type I receptor (TGF*β*R1), which phosphorylates receptor-regulated Smad (R-Smad), Smad2, and Smad3 [36]. In addition, LPA potentially induces the direct phosphorylation of Smad2/3 via LPA receptors, or LPA-induced TGF-*β*1 signals activate phosphorylation of Smad2/3. 

Phosphorylated Smad2/3 forms complex with Smad4 and translocates into the nucleus. It has been reported that smad4 functions as a positive transcriptional regulator of Ln-332 in colorectal cancer cell lines [42]. Through these potential pathways, LPA potentially enhances Ln-332 expression, leading to the promotion of A431 colony dispersal. 

However, we recognize that our results could be the consequences of the activation of other pathways than TGF-*β*. In brief, we acknowledge that many previous studies have explored the downstream effectors of LPA, including RhoA, which is involved in contraction and cell rounding, Rac, which is involved in cell spreading and migration, Akt, which is involved in cell survival, and RAS, which is important in DNA synthesis and viability [51]. These multiple effects may explain why one compound, LPA, could have such a repertoire of cell responses. 

## 4. Conclusions

Herein, we have reported that LPA induces TGF-*β*1 target gene expression including Ln-332 during LPA-induced A431 colony dispersal, and we conclude that Ln-332 plays a significant role in promoting colony dispersal and single-cell migration, at least for the A431 cell line. 

## Figures and Tables

**Table 1:** *Primers used for quantitative RT-PCR. *For RT-PCR, we analyzed samples on an MiQ machine using a FastStart SYBR Green Master Mix, with the following primer sets.

Gene	Primer set	Gene accession number
LAMA3	5′-GGCTCACTCTGTATTGTTGG	NM_000227
	5′-ACAGAGACTGCTTTGGTGTG	
LAMB3	5′-TGATGGACAGGATGAAAGAC	NM_000228
	5′-GGAAGCTGTAGCATCACTTG	
LAMC2	5′-GAAGCCCAGAAGGTTGATAC	NM_005562
	5′-GTGAGTGTTCTGGAGCAAAG	
GAPDH	5′-ATGACATCAAGAAGGTGGTG	NM_002046
	5′-CTGTAGCCAAATTCGTTGTC	
PPIA	5′-CAAATGCTGGACCCAACACA	NM_021130
	5′-TGCCATCCAACCACTCAGTC	
